# Altered Regulation of Contraction-Induced Akt/mTOR/p70S6k Pathway Signaling in Skeletal Muscle of the Obese Zucker Rat

**DOI:** 10.1155/2009/384683

**Published:** 2010-03-30

**Authors:** Anjaiah Katta, Sunil Kakarla, Miaozong Wu, Satyanarayana Paturi, Murali K. Gadde, Ravikumar Arvapalli, Madhukar Kolli, Kevin M. Rice, Eric R. Blough

**Affiliations:** ^1^Department of Pharmacology, Physiology, and Toxicology, Joan C. Edwards School of Medicine, Marshall University, Huntington, WV 25755, USA; ^2^Cell Differentiation and Development Center, Marshall University, Huntington, WV 25755, USA; ^3^Department of Biological Sciences, Marshall University, Huntington, WV 25755, USA; ^4^Laboratory of Molecular Physiology, Marshall University, 241N Byrd Biotechnology Building, 1 John Marshall Drive, Huntington, WV 25755-1090, USA; ^5^Department of Exercise Science, Sport and Recreation, College of Education and Human Services, Marshall University, Huntington WV 25755, USA

## Abstract

Increased muscle loading results in the phosphorylation of the 70 kDa ribosomal S6 kinase (p70S6k), and this event is strongly correlated with the degree of muscle adaptation following resistance exercise. Whether insulin resistance or the comorbidities associated with this disorder may affect the ability of skeletal muscle to activate p70S6k signaling following an exercise stimulus remains unclear. Here, we compare the contraction-induced activation of p70S6k signaling in the plantaris muscles of lean and insulin resistant obese Zucker rats following a single bout of increased contractile loading. Compared to lean animals, the basal phosphorylation of p70S6k (Thr389; 37.2% and Thr421/Ser424; 101.4%), Akt (Thr308; 25.1%), and mTOR (Ser2448; 63.0%) was higher in obese animals. Contraction increased the phosphorylation of p70S6k (Thr389), Akt (Ser473), and mTOR (Ser2448) in both models however the magnitude and kinetics of activation differed between models. These results suggest that contraction-induced activation of p70S6k signaling is altered in the muscle of the insulin resistant obese Zucker rat.

## 1. Introduction

Insulin resistance is an important health concern that is rapidly increasing worldwide. If allowed to precede unchecked, insulin resistance often leads to increased morbidity and mortality. Anaerobic exercise has recently been recognized to be of potential benefit for the treatment of this condition [1–4], as progressive resistance training has been found to improve glycemic control, increase skeletal muscle size and strength, while also decreasing visceral and total body fat [5–7]. Whether insulin resistance affects how muscle responds to resistance training is unknown, but the existence of differences, if present, may help to explain why exercise-induced skeletal muscle adaptations may differ between normal and diabetic muscle [8–10]. 

Recent in vitro and in vivo studies have suggested that increased muscle loading correlates with increases in the rates of accumulation and synthesis of muscle protein [11–14]. This increase in protein synthesis, at least in part, is thought to be regulated by the phosphorylation of the p70 ribosomal protein S6 kinase (p70S6k) [11, 15, 16] and the mammalian target of rapamycin (mTOR) which functions as a growth factor and nutrient-sensing signaling molecule in mammalian cells [17]. How mTOR activity is modulated is not entirely clear; however previous studies have reported that resistance exercise increases mTOR activity via an upstream pathway involving phosphoinositide 3-kinase (PI3K) and protein kinase B (PKB)/Akt [15, 18, 19]. Other work has suggested that the activity of PI3K/Akt signaling pathway can be negatively regulated by the phosphatase and tensin homologue deleted on chromosome 10 (PTEN) [20]. How the activity of these pathways are regulated following increased muscle contraction and if insulin resistance affects these processes are not well understood. 

The purpose of this study was to determine whether exercise-induced p70S6k signaling is altered in the skeletal muscle of the insulin resistant obese Zucker rat. We hypothesized that contraction-induced p70S6k signaling would differ between normal and insulin resistant muscle. To test this hypothesis, the activation of p70S6k and its pathway related proteins mTOR, Akt, and PTEN were assessed in the plantaris muscles of normal and insulin resistance rats either immediately after or during the recovery phase of an acute bout of high-frequency electrical stimulation. Our findings suggest that insulin resistance or the co-morbidities associated with this disorder may affect how muscle regulates contractile-induced signaling.

## 2. Materials and Methods

### 2.1. Animal Care

All procedures were performed as outlined in the Guide for the Care and Use of Laboratory Animals as approved by the Council of the American Physiological Society and the institutional animal use review board. Young (10 week, *n* = 12) male Lean Normal Zucker (LNZ) and young (10 week, *n* = 12) male Obese Syndrome-X Zucker (OSXZ) rats were obtained from the Charles River Laboratories. Rats were housed two to a cage in an AAALAC approved vivarium. Housing conditions consisted of a 12 H: 12 H dark-light cycle and temperature was maintained at 22° ± 2°C. Animals were provided food and water ad libitum and allowed to recover from shipment for at least two weeks before experimentation. During this time the animals were carefully observed and weighed weekly to ensure none exhibited signs of failure to thrive, such as precipitous weight loss, disinterest in the environment, or unexpected gait alterations.

### 2.2. Materials

Anti-p70S6k (#9202), Akt (#9272), mTOR (#2972), glycogen synthase kinase-3*β* (GSK-3*β*) (#9332), PTEN (#9552), Thr389 (#9206) and Ser 421 / Thr 424 (#9204) phosphorylated p70S6K, Thr308 (#9275) and Ser473 (#9271) phosphorylated Akt, Ser2448 phosphorylated mTOR (#2971), Ser 9 phosphorylated GSK-3*β* (#9336), Ser 380/Thr 382/383 phosphorylated PTEN (#9554), Mouse IgG, and Rabbit IgG antibodies were purchased from Cell Signaling Technology (Beverly, MA). Enhanced chemiluminescence (ECL) western blotting detection reagent was from Amersham Biosciences (Piscataway, NJ). Restore western blot stripping buffer was obtained from Pierce (Rockford, IL) and 3T3 cell lysates were from Santa Cruz Biotechnology (Santa Cruz, CA). All other chemicals were purchased from Sigma (St. Louis, MO) or Fisher Scientific (Hanover, IL).

### 2.3. Contractile Stimulation of Skeletal Muscles

The high-frequency electrical stimulation (HFES) model has been previously described [21] and was chosen on the basis of its efficacy in stimulating protein translation and muscle hypertrophy in vivo [11]. The HFES model used in the present study produced 10 sets of 6 contractions with an overall protocol time of 22 minutes. This protocol results in concentric (shortening) contraction of the plantaris and soleus and eccentric (lengthening) contraction of the EDL and TA. Animals were killed by a lethal dose of pentobarbital sodium at baseline, immediately following, 1 or 3 hours after HFES. Once excised, muscles were blotted dry, trimmed of visible fat and tendon projections, weighed, immediately frozen in liquid nitrogen, and stored at −80°C.

### 2.4. Preparation of Protein Isolates and Immunoblotting

Plantaris muscles were pulverized in liquid nitrogen using a mortar and pestle until a fine powder was obtained. After washing with ice cold PBS, pellets were lysed on ice in for 15 minutes in T-PER lysis buffer composed of T-PER reagent (2 mL/g tissue weight) (Pierce, Rockford, IL), 1 mM ethylene-diamine tetraacetic acid (EDTA; pH 8.0), 1 mM ethylene-glycol tetraacetic acid (EGTA; pH 7.5), 1 mM magnesium chloride, phenylmethane sulfonylfluoride (PMSF), 1 *μ*L protease inhibitor cocktail, and 1 mM sodium vanadate to inhibit phosphatase activity, and then centrifuged for 10 minutes at 2000 g to pellet particulate matter. This process was repeated twice and the supernants combined for protein concentration determination using the Bradford method (Pierce, Rockford, IL). Samples were diluted to a concentration of 3 *μ*g/*μ*L in SDS loading buffer, boiled for 5 minutes, and 60 *μ*g of protein were separated using 10% SDS-PAGE gels. Transfer of protein onto nitrocellulose membranes, verification of transfer, and determination of equal loading between lanes and membranes was determined as outlined previously [22]. Protein immunodetection was performed as outlined by the antibody manufacturer while immunoreactive bands were visualized with ECL (Amersham Biosciences). Exposure time was adjusted to keep the integrated optical densities within a linear and nonsaturated range. Band signal intensity was normalized to *β*-actin by densitometry using a flatbed scanner (Epson Perfection 3200 PHOTO) and Imaging software (AlphaEaseFC). Molecular weight markers (Cell Signaling) were used as molecular mass standards and NIH 3T3 cell lysates were included as positive controls. To allow direct comparisons to be made between the concentration levels of different signaling molecules, immunoblots were stripped and reprobed with Restore western blot stripping buffer as detailed by the manufacturer (Pierce, Rockford, IL).

### 2.5. Statistical Analysis

Results are presented as mean ± SEM. Data were analyzed by using the Sigma Stat 3.0 statistical program. The effects of insulin resistance on protein phosphorylation were analyzed using a two-way ANOVA followed by the Student-Newman-Keuls post-hoc testing when appropriate. Differences were considered significant at *P* < .05.

## 3. Results

Average body mass of the obese Zucker rats was 82.0% higher (597 ± 21.7 gm versus 328 ± 12.2 gm) than their lean counterparts (*P* < .05). Plantaris muscle mass in the obese Zucker rat expressed as a percentage of body weight was 35.9% less than that (0.41 ± 0.01 versus 1.14 ± 0.04 mg/gm) seen in the lean Zucker rat (*P*  <  .05). 

### 3.1. Akt-p70S6k Pathway Protein Expression and Level of Basal Phosphorylation is Altered in the Skeletal Muscle of the Obese Zucker Rat

**Figure 1:**
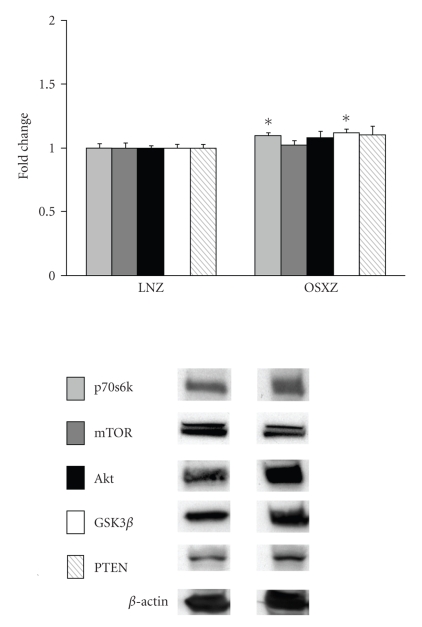
Plantaris muscles from lean Zuckers (LNZ) and obese Zuckers (OSXZ) were analyzed by Western blot analysis for insulin resistance-related changes in total p70S6k, mTOR, Akt, GSK-3*β* and PTEN protein expression. Protein quantification was done after normalization by the abundance of *β*-actin. An asterisk (*) indicates significant differences (*P* < .05) from the lean Zucker value.

**Figure 2:**
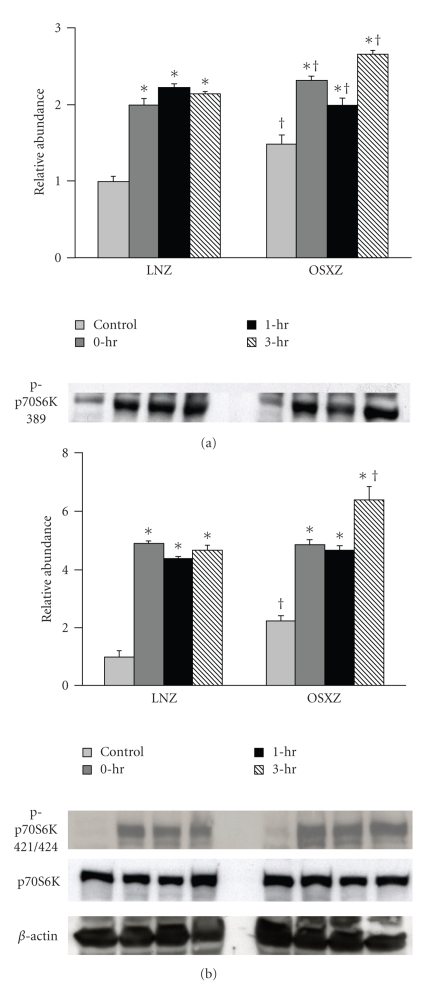
Contraction-induced p70S6k phosphorylation is altered with insulin resistance. The basal (control) and contraction-induced phosphorylation of the p70S6k in Plantaris muscles from lean (LNZ) and obese (OSXZ) Zucker rats at 0, 1, and 3 hours after contractile stimulus. p70S6k (Thr389 & Thr421/Ser424) phosphorylation was determined by immunoblotting for phosphorylation on Thr389 and Thr421/Ser424. Relative abundance represents ratio of phospho to total protein. An asterisk (*) indicates significant difference (*P* < .05) from the control animals and a dagger (^†^) indicates a significant difference (*P* < .05) from the corresponding time points across animal models.

**Figure 3:**
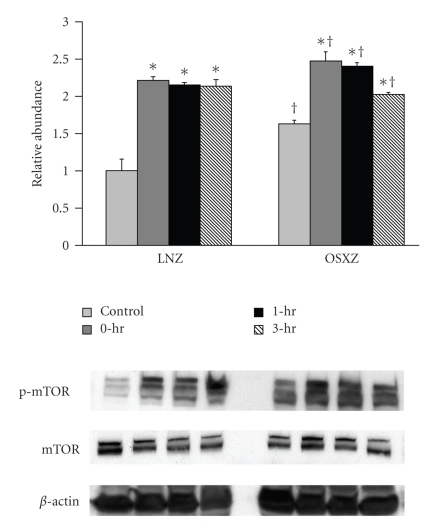
Contraction-induced mTOR (Ser 2448) phosphorylation is altered with insulin resistace. The basal (control) and contraction-induced phosphorylation of the mTOR in Plantaris muscles from lean (LNZ) and obese (OSXZ) Zucker rats at 0, 1, and 3-hours after HFES. Phosphorylation of mTOR was determined by immunoblotting for phosphorylation on Ser2448. Relative abundance represents ratio of phosphor to total protein. An asterisk (*) indicates significant difference (*P* < .05) from the control animals and a dagger (^†^) indicates significant difference (*P* < .05) from corresponding time points across the animal models.

**Figure 4:**
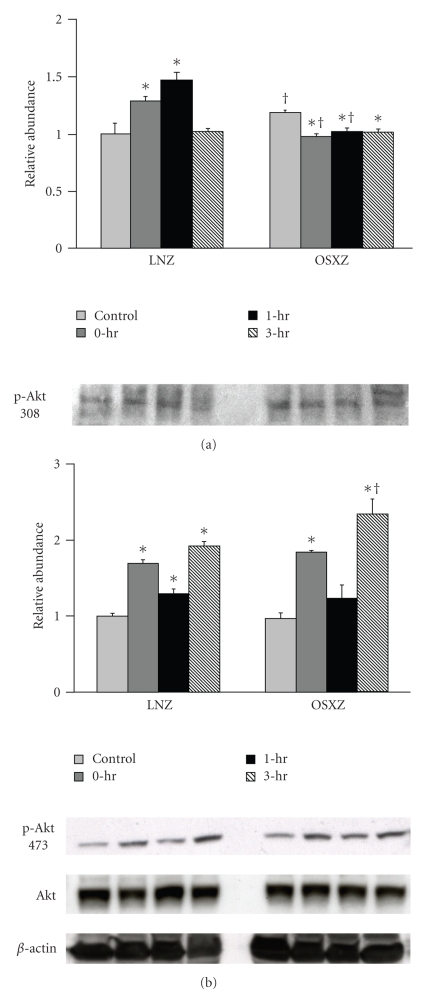
Effects of concentric, maximal muscle contraction in situ on phosphorylation of Akt (Thr308/Ser473). The basal (control) and contraction-induced phosphorylation of the Akt in Plantaris muscles from lean (LNZ) and obese (OSXZ) Zucker rats at 0, 1, and 3 hours after HFES. Akt phosphorylation was determined by immunoblotting for Akt phosphorylation on Thr308 and Ser473. Relative abundance represents ratio of phosphor to total protein. An asterisk (*) indicates significant difference (*P* < .05) from the control n animals, and a dagger (^†^) indicates significant difference (*P* < .05) from corresponding time points across the animal models.

**Figure 5:**
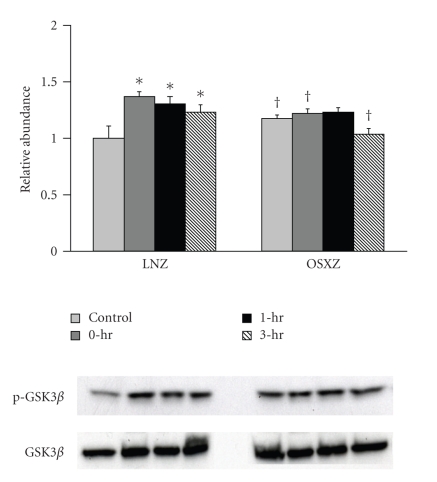
Contraction-induced GSK-3*β* (Ser 9) phosphorylation is altered with insulin resistance. The basal (control) and contraction-induced phosphorylation of the GSK-3*β* in Plantaris muscles from lean (LNZ) and obese (OSXZ) Zucker rats at 0-, 1-, and 3-hours after HFES. Phosphorylation of GSK-3*β* was determined by immunodetection of phosphorylation on Ser 9. Relative abundance represents ratio of phosphor to total protein. An asterisk (*) indicates significant difference (*P* < .05) from the control animals, and a dagger (^†^) indicates significant difference (*P* < .05) from corresponding time points across the animal models.

**Figure 6:**
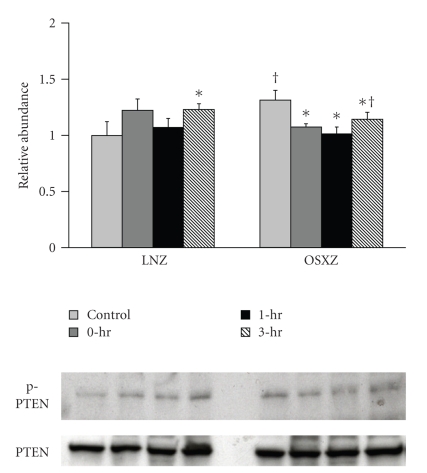
Contraction-induced PTEN (Ser380/Thr382/383) phosphorylation is altered with insulin resistance. The basal (control) and contraction-induced phosphorylation of the GSK-3*β* in Plantaris and soleus muscles from lean (LNZ) and obese Zucker (OSXZ) rats at 0-, 1-, and 3-hours after HFES. Phosphorylation of PTEN was determined by immunodetection of phosphorylation on Ser380/Thr382/383. Relative abundance represents ratio of phosphor to total protein. An asterisk (*) indicates significant difference (*P* < .05) from the control time point within animal model, and a cross (^†^) indicates significant difference (*P* < .05) at corresponding time points across animal models.

Immunoblotting analysis demonstrated that the expression levels of p70S6k and GSK-3*β* were ~10% and ~12% higher in the obese Zucker plantaris compared to muscles obtained from the lean Zucker (*P* <  .05) (Figure 1). There were not significant differences in Akt, mTOR or PTEN protein content (Figure 1). As p70S6k, mTOR, Akt, GSK-3*β*, and PTEN are thought to be regulated by phosphorylation; it was of interest to determine if the basal phosphorylation status of these proteins is altered with insulin resistance. Immunoblotting using phospho-specific antibodies indicated that, the basal level phosphorylation of p70S6k (Thr389) and p70S6k (Thr421/Ser424) was 37.2% and 101.4% higher, respectively, in the obese Zucker plantaris (*P*  <  .05) (Figure 2). Similarly, the basal level phosphorylation of mTOR (Ser 2448), Akt (Ser 308), GSK-3*β* (Ser 9), and PTEN (Ser 380/Thr 382/383) was 63.0%, 25.1%, 17.5% and 31.4% higher, respectively, with insulin resistance (*P*  <  .05) (Figures 3–6). Akt (Ser473) phosphorylation was not different with insulin resistance (Figure 4).

### 3.2. Regulation of Akt-p70S6k Signaling in Response to a Contractile Stimulus Is Altered in the Skeletal Muscle of the Obese Zucker Rat

Phosphorylation of p70S6k, mTOR, Akt, PTEN, and GSK-3*β* in exercised plantaris muscles was determined at 0, 1, and 3 hours after a bout of HFES and compared with control (unstimulated) muscles. Contraction-induced phosphorylation of these molecules was compared between lean and obese Zucker rats. In the case of each molecule examined, significant differences existed between lean and obese Zucker rat models (Figures 2–6). In the lean Zucker, p70S6k (Thr389) phosphorylation increased 112.8%, 130.0%, and 96.2% while the phosphorylation of p70S6k (Thr 421/Ser424) increased 429.8%, 354.5%, and 319.2% at 0, 1, and 3 hour post exercise respectively (*P* < .05). In the obese Zucker, HFES increased p70S6k (Thr389) phosphorylation 79.9%, 43.6% and 104.5% and the phosphorylation of p70S6k (Thr 421/Ser 424) by 158.9%, 127.8%, and 238.6% at 0, 1, and 3 hours post exercise, respectively, (*P* < .05) (Figure 2). In the lean Zucker animals, the phosphorylation level of mTOR (Ser2448) was increased 121.7%, 115.5%, and 113.8%, from baseline at 0, 1 and 3 hours postexercise, respectively (*P* < .05) (Figure 3) whereas mTOR (Ser 2448) phosphorylation in the obese animals was increased by 51.8%, 47.4%, and 24.5% during the same time periods (*P* < .05) (Figure 3). 

Lean animals increased the phosphorylation of Akt (Thr308) by 59.9% and 40.8% immediately after and 1 hour post exercise while Akt (Ser473) was increased by 109.9%, 23.7%, and 105.9% at 0, 1, and 3 hours post exercise, respectively, (*P* < .05) (Figure 4). Conversely, in the obese animals, Akt (Thr308) phoshorylation was decreased by 21.5%, 18.8%, and 15.3% at 0, 1, and 3 hours post exercise while the phosphorylation of Akt (Ser473) was increased by 81.8% and 140.9% at 0, and 3 hours post exercise (*P*  <  .05) (Figure 4). 

Confirming these differences in Akt phosphorylation, HFES increased GSK-3*β* (Ser 9) phosphorylation in the lean animals by 36.9%, 30.4%, and 23.4% at 0, 1, and 3 hours post exercise (*P* < .05) (Figure 5). GSK-3*β* (Ser 9) phosphorylation did not change with contraction in the obese plantaris. In lean animals, PTEN (Ser380/Thr382/383) phosphorylation was increased 23.1% at 3 hours post exercise while conversely, in the obese Zucker animals, PTEN phosphorylation decreased by 18.3%, 23.0%, and 13.1% at 0, 1 and 3 hours post exercise, respectively (*P* < .05) (Figure 6).

## 4. Discussion

The obese Zucker (*fa*/*fa*) rat is a well-established animal model of skeletal muscle insulin resistance that is characterized by marked hyperinsulinemia, glucose intolerance, dyslipidemia, and central adiposity. Recent data has suggested that normal and insulin resistant muscle may differ in their adaptation to an exercise regimen [1, 8, 10, 23–25]. The molecular mechanism(s) responsible for these differences have not been widely studied. Here we examine if the contractile-induced muscle signaling altered in the obese Zucker rat. Taken together, our data suggests that insulin resistance may be associated with differences in how skeletal muscle regulates Akt-p70S6k signaling following an acute bout of high intensity muscle contraction. 

### 4.1. Insulin Resistance Muscle Is Associated with Alterations in Akt Pathway Protein Content and Basal Phosphorylation

Similar to previous work using obese rats [26] and mice [27], we demonstrate that insulin resistant muscle is associated with differences in how the p70S6k signaling pathway is regulated. Specifically, we found that the basal phosphorylation level of p70S6k, mTOR, Akt, and GSK-3*β* was higher in the insulin resistant plantaris muscles of obese rats compared to that found in their lean counterparts (Figures 2–5). Previous data has suggested that hyperinsulinemia and/or hyperglycemia may be associated with increased Akt protein and Akt pathway activation [28–31]. For example, recent work has reported that the obese Zucker kidney exhibits increased Akt kinase activity and expression of phosphorylated Akt and mTOR compared to that observed in lean Zucker animals [32]. Increased circulating amino acid levels have also been shown to activate mTOR [29, 33]. Whether elevated insulin or amino acids are responsible for the changes we observe in the present study is unknown. Further experimentation perhaps employing other methods of analysis or approaches is necessary to better understand how changes in the basal phosphorylation of p70S6k, mTOR, Akt, and GSK-3*β* may contribute to pathophysiology of insulin resistance.

### 4.2. Insulin Resistance is Associated with Alteration in the Contraction-Induced Activation of Akt Signaling

Previous work has demonstrated that resistance exercise is capable of increasing the phosphorylation (activation) of the p70S6k [11]. Activated p70S6k in turn, is thought to phosphorylate the ribosomal protein S6 enabling the up-regulation of 5′ TOP mRNA for encoding translational machinery and ribosomal proteins [34]. Here we report that the magnitude and time course of the contraction-induced phosphorylation of p70S6k (Thr 389) and p70S6k (Thr 421/Ser 424) appears to be significantly different in obese Zucker plantaris muscle compared to that observed in the normal plantaris (Figure 2). To our knowledge, this finding has not been reported before. The underlying molecular mechanisms for these alterations remains unclear; however it is interesting to note that previous reports have suggested that the insulin-stimulated phosphorylation of p70S6k may be altered in diabetic rats [35–37]. In the light of these studies, our data suggest that insulin resistance may affect how multiple stimuli may regulate the phosphorylation of p70S6k. Future studies designed to examine if insulin resistance affects the regulation of p70S6k using different modes of contraction, different intensities of exercise, or muscles with different muscle fiber types will no doubt be useful in furthering our understanding of the effects of insulin resistance on p70S6k activation.

Similar to previous studies, increased contractile activity appears to be a strong stimulus to increase the phosphorylation level of mTOR in nondiabetic muscle [21, 38], however the magnitude and time course of the contraction-induced phosphorylation of mTOR was significantly different in plantaris muscles of the obese (insulin resistant) animals (Figure 3). This latter finding is consistent with our data demonstrating that diabetic muscle exhibits a reduced ability to activate p70S6k following a bout of HFES. Like p70S6k, mTOR is thought to be involved in the regulating several components of the translational machinery, and in addition is thought to be an upstream activator of p70S6k [39]. The factor(s) regulating mTOR are not fully understood. mTOR has been shown to phosphorylate p70S6k in vitro and it is thought that this occurs via a mechanism distinct from the PI3K-dependent pathway [40–42]. Indeed, recent studies have shown activation of mTOR/p70S6k even in the absence of Akt activation [42–44]. Although not investigated here, it has been suggested that phospholipase D (PLD) may play a key role in regulating mTOR phosphorylation in response to increased muscle loading [40, 41, 45, 46]. Whether differences in PLD-dependent signaling following contractile loading exist between the lean and obese Zucker animals is currently unknown. Nonetheless, our findings are consistent with the notion that insulin resistance associated alterations in p70S6k regulation may be due, at least in part, to defects in the ability of insulin resistant muscle to activate mTOR following a contractile stimulus. 

The precise influence of insulin resistance on Akt regulation in muscle contraction remains unclear. Akt is a critical signaling mediator of cellular growth and metabolism in skeletal muscle [15], that is, translocated to the muscle membrane when activated by phosphorylation at the Thr308 and Ser 473 residues [47]. Phosphorylation at both these sites is critical for full activation of Akt. The phosphorylation of Thr-308 is apparently catalyzed by a 3-phosphoinositide-dependent protein kinase (PDK1), which is active only in the presence of phosphatidylinositol-3,4,5-triphosphate and phosphatidylinositol-3,4-biphosphate [48]. Similar to others [49–51], we found that a bout of HFES significantly increased the phosphorylation of both Akt (Thr 308) and Akt (Ser 473) in plantaris muscles of the lean Zucker rats (Figure 4). Conversely, in the insulin resistant plantaris, HFES appeared to only increase the phosphorylation of the Akt Ser 473 (Figure 4). The activation of Akt is thought to be a multistep process and is likely one that involved several proteins [52]. In an effort to determine a possible mechanism for the alterations in Akt regulation we observed in the obese Zucker plantaris muscle we next examined the effects of HFES on the Akt inhibitor PTEN. It is thought that phosphorylation of PTEN inhibits its phosphatase activity [20]. As expected from our analysis of how insulin resistance affected the basal phosophorylation of p70S6k, mTOR and Akt, we found a higher level of basal PTEN phosphorylation in the obese Zucker plantaris muscle compared to that observed in lean animals (Figure 6). Furthermore, with contraction we found a significant decrease in the phosphorylation of PTEN in the plantaris muscles obtained from the obese Zucker animals. Whether this decrease in PTEN phosphorylation we see with contraction is sufficient to explain the differences in Akt phosphorylation we observe in the insulin resistant plantaris is currently unknown. Similarly, it should be noted that the functional role of PTEN in regulating Akt activation cannot be accurately assessed in the absence of further study to evaluate the experimental manipulation of this protein. Additional studies perhaps employing strategies designed to directly inhibit or activate PTEN during HFES may prove useful in addressing these possibilities.

To explore Akt-related signaling further, we next investigated the regulation of Akt substrate GSK-3*β*. Similar to our findings concerning Akt at Thr 308 phosphorylation, we failed to find any increase in GSK-3*β* (Ser 9) phosphorylation with HFES in the obese Zucker plantaris. The significance of this finding is currently not known, however it is thought that the Akt-dependent phosphorylation of GSK-3*β* leads to the activation of pathways promoting protein synthesis via a decreased inhibition of the translational initiation factor eIF2B [53]. Taken together, these data suggests that insulin resistance may be associated with alterations in the ability of fast twitch muscle to activate Akt. Why the regulation of Akt following HFES may differ in the obese Zucker rat is not known but this change may be due to defects in the ability of insulin to activate PI3K signaling in response to increased muscle loading. Alternatively, it has been postulated that the degree of Akt activation following contractile activity may be dependent upon the type of contractile activity, contraction intensity, and/or the duration of stimulation [21]. Given this contention, it is possible, that differences in the signaling response between models could be related to the time points chosen for evaluation. Similarly, it should be noted that although the magnitude or time course of contraction-induced signaling may differ between experimental groups, the degree of overall activation postexercise was similar in both groups for most of the Akt signaling protein examined. As such it is possible that the differences in magnitude of contraction-induced “activation” we observed may be simply attributed to differences in the initial level of basal phosphorylation. A future study employing other time points or experimental approaches is certainly warranted. 

In summary, our data are consistent with the notion that the insulin resistance may alter contractile signal transduction pathways in skeletal muscle. Whether this finding alone is able to explain differences in how insulin resistant muscle respond to exercise regimen is not known and beyond the scope of this study. Likewise, whether the differences we observe between models is due to insulin resistance or to the existence of other abnormalities associated with the obese phenotype or other characteristics of the obese Zucker rat model is currently unclear. Given the multitude of factors thought to be involved in regulating how skeletal muscle “senses” and “responds” to increased loading stimuli (e.g., muscle hypoxia, reactive oxygen levels, muscle damage, local release of growth factors, and cytokines [54]) it will be interesting what further research, perhaps using in vitro contraction paradigms will add to our understanding of the interplay between insulin resistance and contraction-induced muscle signaling.
